# Understanding Dementia Caregiver Needs: A Qualitative Analysis of National Helpline Calls

**DOI:** 10.1093/geroni/igaf122.4038

**Published:** 2025-12-31

**Authors:** Nancy Hodgson, Sonia Talwar, Kerry Finegan, Liming Huang, Todd Myers

**Affiliations:** University of Pennsylvania, Philadelphia, Pennsylvania, United States; University of Pennsylvania, Philadelphia, Pennsylvania, United States; Alzheimer’s Association, Chicago, Illinois, United States; University of Pennsylvania, Philadelphia, Pennsylvania, United States; University of Pennsylvania, Philadelphia, Pennsylvania, United States

## Abstract

Over 11 million Americans provide unpaid care to persons living with dementia (PLWD), leading to social isolation, financial hardship, and increased risk for depression and stress. Despite available resources, dementia caregivers frequently lack access to coordinated support. Telephone support programs offer accessible interventions, but little is known about specific needs caregivers express when seeking help. This study examined challenges and needs reported by callers to the Alzheimer’s Association National Helpline (AANH). We completed a qualitative content analysis of 1,000 call logs from the AANH (July 2021 to July 2022). Three researchers independently coded call logs using open coding followed by development of a refined 21-code framework. Caregiver-presenting challenges and needs were identified, categorized, and ranked by frequency. The average Kappa value among coders was 0.47, with 72% agreement. The ten most frequently reported needs were: home and community-based services (19%), communication techniques with PLWD (16%), caregiver self-care and emotional support (15%), managing neuropsychiatric symptoms and behaviors (10%), medical management (9%), relevant educational resources (7%), legal resources (5%), insurance and healthcare financing (5%), home safety resources (5%), and transition to long-term care advice (5%). Most caregivers were women, White, caring for spouses, and over 65 years old. This newly completed analysis reveals that dementia caregivers most frequently sought guidance for concerns routinely not discussed during healthcare encounters. These fresh findings highlight critical gaps in current care delivery, offering opportunities to better support this vulnerable population through proactive assessment by healthcare providers, the development of tailored interventions, and policy efforts.

